# Intermedin inhibits unilateral ureteral obstruction-induced oxidative stress via NADPH oxidase Nox4 and cAMP-dependent mechanisms

**DOI:** 10.1080/0886022X.2017.1361839

**Published:** 2017-08-14

**Authors:** Xi Qiao, Lihua Wang, Yanhong Wang, Xiaole Su, Yue Qi, Yun Fan, Zhiqiang Peng

**Affiliations:** a Department of Nephrology, Second Hospital of Shanxi Medical University, Shanxi, China;; b Shanxi Kidney Disease Institute, Shanxi, China;; c Department of Microbiology and Immunology, Shanxi Medical University, Taiyuan, Shanxi, China

**Keywords:** Intermedin, NADPH oxidase, reactive oxygen species, cAMP, renal

## Abstract

NADPH oxidase Nox4-derived reactive oxygen species (ROS) play important roles in renal fibrosis. Our previous study demonstrated that intermedin (IMD) alleviated unilateral ureteral obstruction (UUO)-induced renal fibrosis by inhibition of ROS. However, the precise mechanisms remain unclear. Herein, we investigated the effect of IMD on Nox4 expression and NADPH oxidase activity in rat UUO model, and explored if these effect were achieved through cAMP-PKA pathway, the important post-receptor signal transduction pathway of IMD, in TGF-β1-stimulated rat proximal tubular cell (NRK-52E). Renal fibrosis was induced by UUO. NRK-52E was exposed to rhTGF-β1 to establish an *in vitro* model of fibrosis. IMD was overexpressed in the kidney and in NRK-52E by IMD gene transfer. We studied UUO-induced ROS by measuring dihydroethidium levels and lipid peroxidation end-product 4-hydroxynonenal expression. Nox4 expression in the obstructed kidney of UUO rat or in TGF-β1-stimulated NRK-52E was measured by quantitative RT-PCR and Western blotting. We analyzed NADPH oxidase activity using a lucigenin-enhanced chemiluminescence system. We showed that UUO-stimulated ROS production was remarkably attenuated by IMD gene transfer. IMD overexpression inhibited UUO-induced up-regulation of Nox4 and activation of NADPH oxidase. Consistent with *in vivo* results, TGF-β1-stimulated increase in Nox4 expression and NADPH oxidase activity was blocked by IMD. In NRK-52E, these beneficial effects of IMD were abolished by pretreatment with *N*-[2-(*p*-bromocinnamylamino)ethyl]-5-isoquinolinesulfonamide hydrochloride (H-89), a PKA inhibitor, and mimicked by a cell-permeable cAMP analog dibutyl-cAMP. Our results indicate that IMD exerts anti-oxidant effects by inhibition of Nox4, and the effect can be mediated by cAMP-PKA pathway.

## Background

Chronic kidney disease (CKD) is one of the fast growing causes of death worldwide [1]. Renal fibrosis plays important roles in the progression of CKD regardless of their etiology [2]. Therefore, preventing renal fibrosis is of great importance for inhibiting the progression of CKD. Reactive oxygen species (ROS) are a group of active intermediate oxygen molecules. Although low levels of ROS are necessary for physiological processes, excessive production leads to oxidative stress, which plays extremely important roles in renal fibrosis [3]. Intermedin [IMD, adrenomedullin-2 (ADM-2)] is a small peptide belonging to the calcitonin/calcitonin gene-related peptide family [4]. We previously reported that IMD alleviates unilateral ureteral obstruction (UUO)-induced renal fibrosis by inhibition of ROS [5]. However, the precise mechanisms remain unknown.

Of the many enzymatic systems in ROS generation in the kidney, nicotinamide adenine dinucleotide phosphate (NADPH) oxidase (Nox) appears to be particularly important [6]. It has been reported that suppressing the production of ROS induced by NADPH oxidase activation inhibits renal fibrosis [7]. There are seven NADPH oxidase isoforms, named Nox1–5, Duox1 and Duox2. Out of them, Nox1, Nox2, and Nox4 are found in the kidney [8]. Nox4 is predominantly localized in renal tubular cell [9]. It has been reported that Nox4 is the main source of ROS in the kidneys during renal fibrosis [10,11].

Previous study demonstrated that ADM blocks angiotensin II-stimulated ROS generation from NADPH oxidase via a cyclic adenosine 3′,5′-monophosphate (cAMP)-protein kinase A (PKA)-dependent mechanism in rat endothelial cells [12]. Whether IMD has any effect on Nox has not yet been clearly described. Since IMD and ADM have similar biological actions [4], we hypothesized that IMD may suppress the production of ROS via NADPH oxidase Nox4 and cAMP-dependent mechanisms. We initially tested the effect of kidney-specific IMD gene transfer on ROS generation in rat UUO model. Next, we examined the influence of IMD overexpression on Nox4 expression and NADPH oxidase activity. Further, we assessed the role that cAMP-PKA plays in the regulation activity of IMD on Nox4 expression and NADPH oxidase activity by using TGF-β1-stimulated rat tubular epithelial cell NRK-52E.

## Materials and methods

### Experimental animals

All animal studies were approved by the Experimental Animal Committee of Shanxi Medical University. Healthy male Wistar rats (∼180 g) were obtained from the Experimental Animal Center of Shanxi Medical University (Taiyuan, China). They were housed under standard laboratory environment and were allowed unlimited access to food and water.

### Preparation of renal fibrosis models

The experimental model of renal fibrosis was established by UUO as we previously reported [5]. Briefly, after giving anesthesia, the left ureter was exposed via a flank incision and ligated at two points with silk. Sham-operated animals were subjected to the exact same surgical procedure, aside from the ureter ligation. Animals were sacrificed on day 7 after operation.

### Overexpression of IMD by gene delivery *in vivo*

Before the left ureter was ligated, pcDNA3.1-IMD plasmid containing full-length complementary DNA (cDNA) sequence of rat IMD or control empty vector pcDNA3.1 was transfected into the kidney using an ultrasound-microbubble mediated method established in our lab [13]. The efficiency of gene transfer was determined by real-time PCR and Western blot analysis.

### Cell culture and transfection

NRK-52E, the rat proximal tubular cell line, was purchased from the Cell Bank of the Chinese Academy of Sciences (Shanghai, China). Cells were grown in Dulbecco’s modified Eagle’s medium (DMEM)/F12 supplemented with 10% fetal bovine serum. Cells were then cultured in fibronectin pre-coated flasks until they reached 70–80% confluence. IMD was over-expressed in NRK-52E by gene transfer with pcDNA3.1-IMD as we previously described [14]. Cell fibrosis model was induced by stimulating with recombinant human TGF-β1 (rhTGF-β1, 10 ng/ml, Gibco, Carlsbad, CA) for 72 h. The cells were randomly allocated into 1 of the following six groups: control (untreated cells); TGF-β1 (cells were stimulated with rhTGF-β1); TGF-β1 + empty vector (cells were transfected with the control plasmid pcDNA3.1 and stimulated with rhTGF-β1); TGF-β1 + IMD (cells were transfected with pcDNA3.1-IMD and stimulated with rhTGF-β1); TGF-β1 + dibutyl-cAMP [cells were stimulated with rhTGF-β1 plus cAMP analog dibutyl-cAMP (10^−3 ^mol/l, Sigma-Aldrich, St. Louis, MO)]; and TGF-β1 + IMD + H-89 [cells were transfected with pcDNA3.1-IMD and stimulated with rhTGF-β1 plus PKA inhibitor H-89 (10^−5 ^mol/l, Sigma-Aldrich, St. Louis, MO)].

### RNA isolation and quantitative real-time PCR

Total mRNA was extracted from the kidney or NRK-52E using Trizol reagent (Invitrogen, Carlsbad, CA), and converted to cDNA (TAKARA, Dalian, China). Quantitative real-time PCR was performed as we previous reported [5]. PCR was performed using the following primers: IMD (sense) 5′-GGCCCAGTTGCTGATGGT-3′ and (antisense) 5′-TGCCCGGGAGCAGGTA-3′, Nox4 (sense) 5′-TTCTGGACCTTTGTGCCTATA C-3′ and (antisense) 5′-CCATGACATCTGAGGGATGATT-3′, β-actin (sense) 5′-CCCATCTATGAGGGTTACGC-3′ and (antisense) 5′-TTTAATGTCACGCAC GATTTC-3′. Each gene was analyzed in triplicate and the expression was normalized to the reference gene β-actin.

### Western blot analyses

Proteins were extracted from kidney or NRK-52E with RIPA lysis buffer and were analyzed by Western blot as we previously described [5]. Briefly, samples were subjected on SDS-PAGE electrophoresis and transferred to PVDF membranes. For immunodetection, membranes were blocked overnight at 4 °C in 5% skim milk and incubated for 2 h at room temperature with the primary antibody rabbit polyclonal anti-IMD (Beijing Biosynthesis Biotechnology, Beijing, China), or rabbit polyclonal anti-Nox4 antibody (Santa Cruz Biotechnology, Santa Cruz, CA). The membranes were then washed with 1 × TBST for three times and incubated with horseradish peroxidase-conjugated goat anti-rabbit antibody (Santa Cruz Biotechnology, Santa Cruz, CA). The specific proteins were detected using an enhanced chemiluminescence (ECL) Western blotting kit (Santa Cruz Biotechnology, Santa Cruz, CA) according to the instructions from the manufacturer. Rabbit polyclonal anti-β-actin (Santa Cruz Biotechnology, Santa Cruz, CA) was served as an internal control.

### Superoxide formation in the kidney

The production of superoxide in the kidney was measured by detecting dihydroethidium (DHE) levels. Briefly, 20 μl of protein lysate was placed in 96-well plates; 200 μl of 10 μM DHE (Sigma-Aldrich, St. Louis, MO) was added to 96-well plates containing 20 μl of kidney lysates. For the measurement of intensity, a fluorescence spectrometer (37 °C) was read for 10 min at excitation/emission filters of 544 nm/612 nm. The control was determined by DHE alone. DHE intensity represented relative fluorescence units (RFUs) per milligram of protein.

### Immunofluorescence

Frozen kidney samples were cut into 7 µm sections and fixed with acetone at −20 °C for 10 min and blocked with 2% bovine serum albumin (BSA) in PBS (1 h, room temperature). Tissue was then incubated with primary antibodies against 4-hydroxynonenal (4-HNE, Abcam, Cambridge, UK) in 2% BSA in PBS for 1 h at room temperature. Secondary fluorescent-conjugated anti-rabbit Alexa Fluor^®^647 (1:400; Invitrogen, Carlsbad, CA) was used for 40 min at room temperature in dark, followed by washing in PBS and distilled water and counterstaining with 4′,6-diamidino-2 phenylindole (DAPI; Invitrogen, Carlsbad, CA) for 5 min. Sections were then washed in PBS and distilled water before mounting with fluorescence mounting medium (Dako, Glostrup, Denmark). Images were obtained using Olympus FV1000 Confocal microscope at ×40 magnification (Olympus Corp., Tokyo, Japan).

### NADPH oxidase assay

We detected NADPH oxidase activity using the lucigenin-enhanced chemiluminescence assay as reported [15]. Homogenates of kidney tissue or NRK-52E were prepared in lysis buffer containing KH_2_PO_4_ (20 mM, pH 7.0), EGTA (1 mM), phenylmethylsulfonyl fluoride (1 mM), aprotinin (10 μg/ml), and leupeptin (0.5 μg/ml). Protein content was estimated using BCA protein assay kit (Beyotime Institute of Biotechnology, Shanghai, China). NADPH activity was measured using an NADPH Activity Quantification Kit (Genmed Scientifics Inc., Shanghai, China) and was expressed as the rate of relative chemiluminescence units per milligram of protein per min.

### Statistical analyses

Data are expressed as mean ± standard deviation (SD). ANOVA and unpaired Student’s *t*-test were performed to analyze for statistical differences between groups. Differences with *p* values of .05 or lower were considered statistically significant.

## Results

### IMD is successfully transfected into the kidney or into NRK-52E

**Figure 1.**
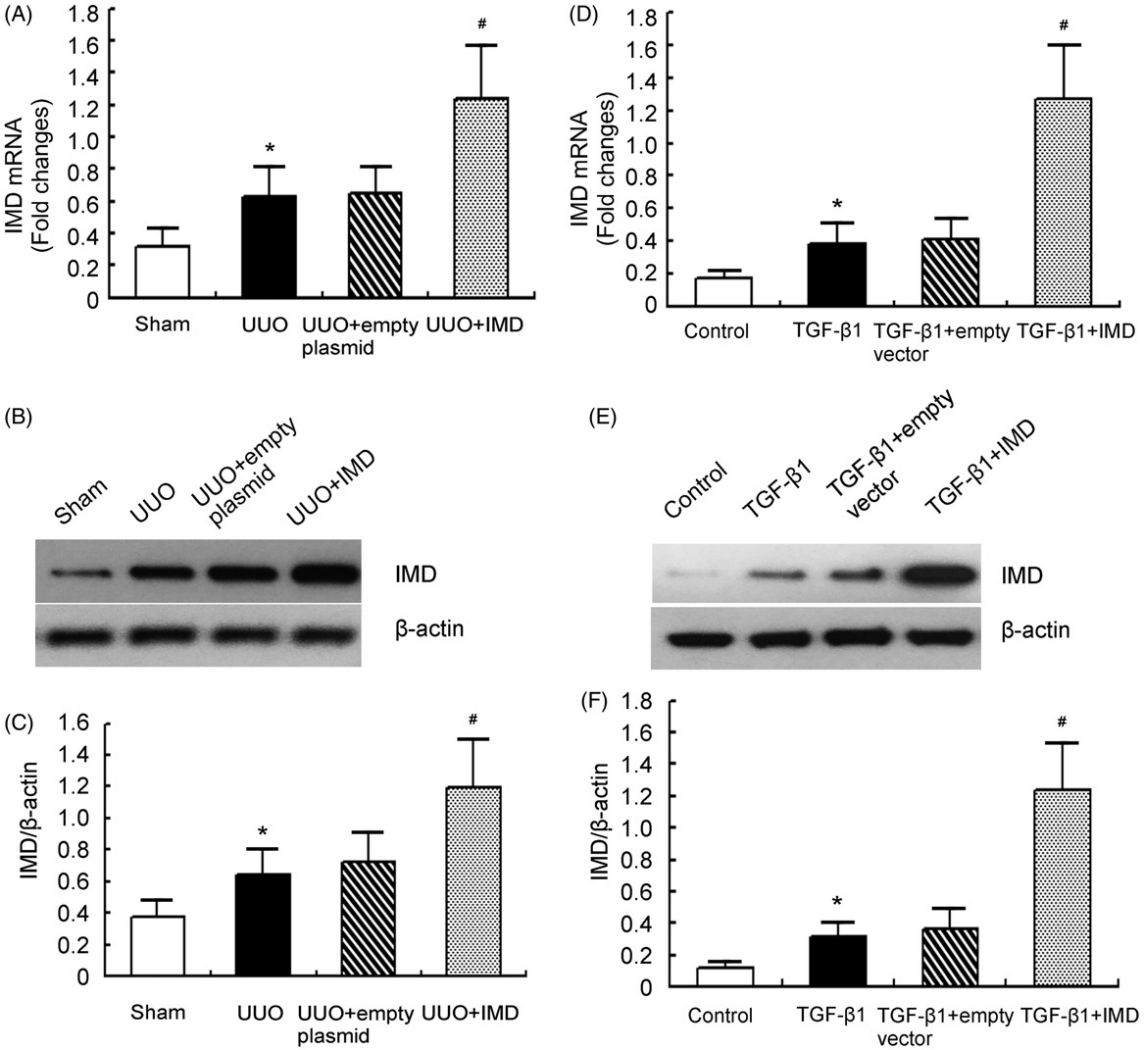
The transfection efficiency of IMD *in vivo* and *in vitro*. (A) IMD mRNA expression measured by quantitative RT-PCR in the obstructed kidney of UUO rats. (B) Representative IMD protein expression measured by Western blot in the obstructed kidney of UUO rats. (C) Densitometric quantifications of band intensities from Western blot for IMD/β-actin in the obstructed kidney of UUO rats. (D) IMD mRNA expression measured by quantitative RT-PCR in NRK-52E cells. (E) Representative IMD protein expression measured by Western blot in NRK-52E cells. (F) Densitometric quantifications of band intensities from Western blot for IMD/β-actin in NRK-52E cells. Data in bar graphs are means ± SD, *n* = 6. *p < .05 versusthe sham (control) group; #*p* < .05 versus the empty vector (empty plasmid) group.

We showed that 7 d after ultrasound-based gene transfer, IMD was significantly overexpressed in the obstructed kidney of pcDNA3.1-IMD treated UUO rats compared with control plasmid treated ones (Figure 1(A–C)). Similarly, *in vitro* study demonstrated that IMD expression was markedly up-regulated by IMD gene transfer (Figure 1(D–F)). These results indicate that IMD is successfully transfected into the kidney or NRK-52E.

### IMD counteracts ROS production in UUO kidneys

**Figure 2.**
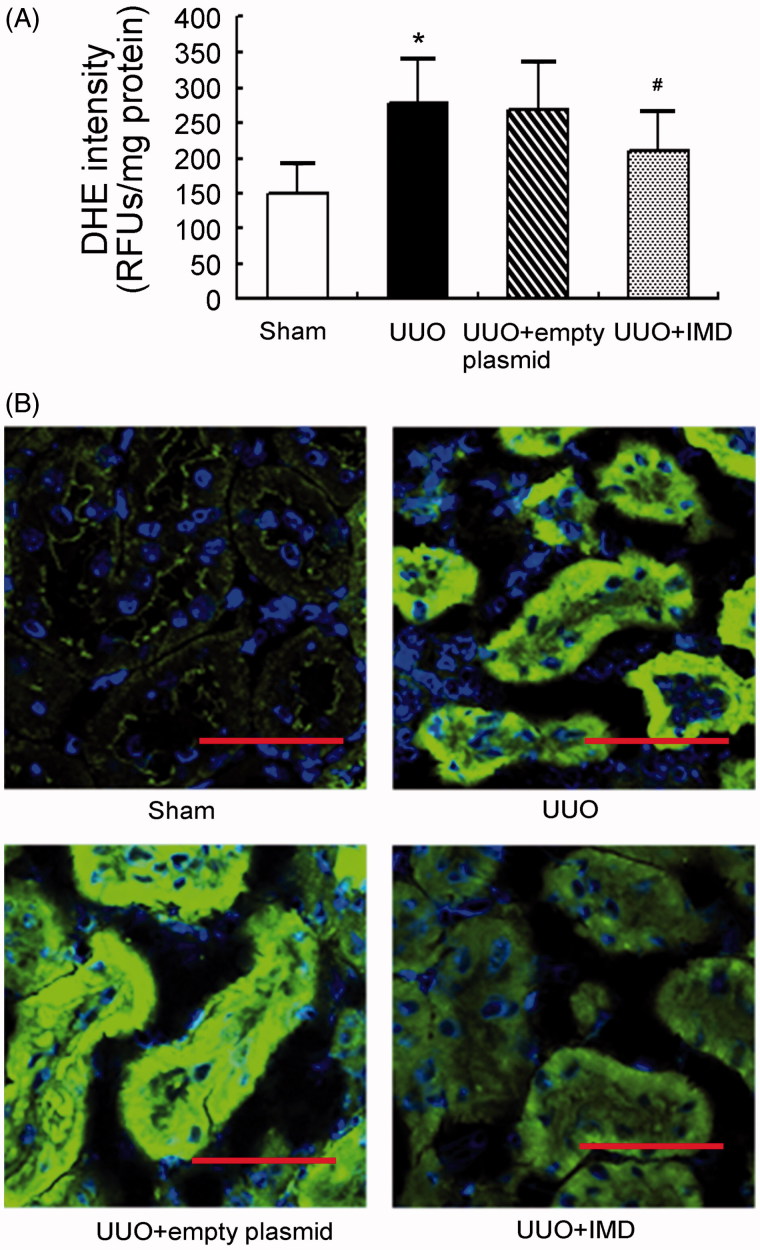
IMD inhibits ROS production in the kidney after UUO. (A) The level of tubulointerstitial superoxides measured by DHE staining. Data in bar graphs are means ± SD, *n* = 6. *p < .05 versus the sham control group; #*p* < .05 versus the UUO group. (B) Respective immunofluorescence staining of 4-hydroxynonenal (4-HNE). Original magnification, ×400. Scale bars: 50 μm.

UUO is known to induce ROS production in the kidney. DHE is widely used for detection of ROS. In our study, a massive increase in DHE generation was identified in the obstructed kidney of UUO rats. It was attenuated by IMD gene delivery, indicating that IMD lowered ROS production in the obstructed kidneys (Figure 2(A)). Supporting these findings, lipid peroxidation (4-HNE staining) was dramatically elevated in the obstructed kidney of UUO animals but significantly reduced by IMD overexpression (Figure 2(B)). By contrast, DHE generation and 4-HNE staining showed no marked differences between obstructed kidneys treated with empty plasmid and non-transfected UUO controls. These results confirm that IMD treatment effectively attenuated ROS production in the kidneys.

### IMD reduces Nox4 expression induced by UUO

**Figure 3.**
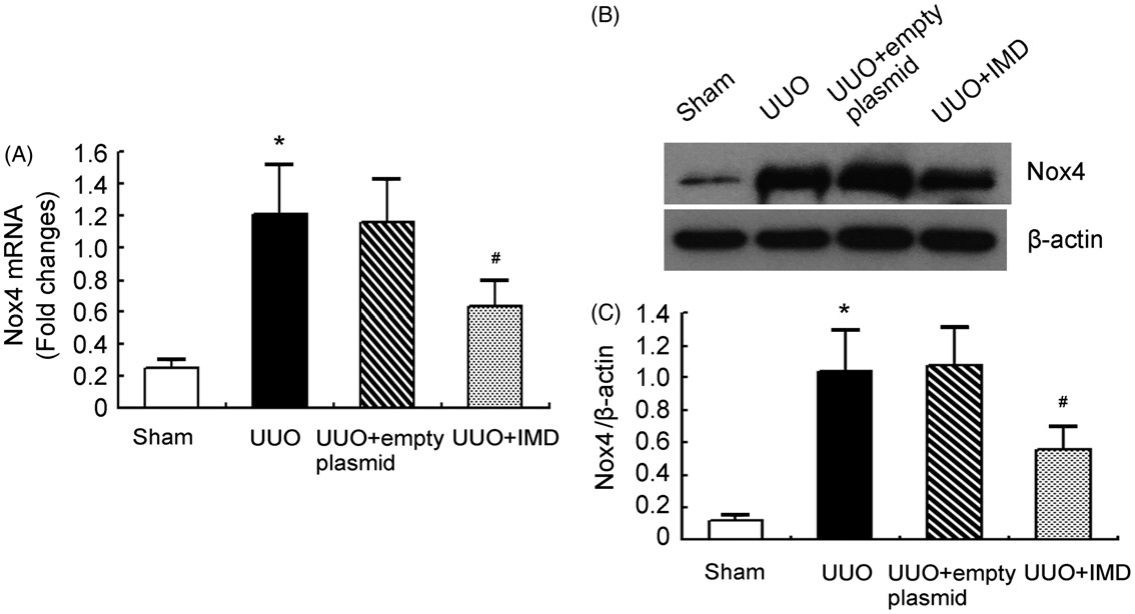
IMD inhibits NADPH oxidase Nox4 expression induced by UUO. (A) Nox4 mRNA expression measured by quantitative RT-PCR in the obstructed kidney of UUO rats. (B) Representative Nox4 protein expression measured by Western blot in the obstructed kidney of UUO rats. (C) Densitometric quantifications of band intensities from Western blot for Nox4/β-actin in the obstructed kidney of UUO rats. Data in bar graphs are means ± SD, *n* = 6. *p < .05 versus the sham control group; #*p* < .05 versus the UUO group.

We determined the influence of IMD on ROS generation system. It is commonly accepted that Nox4 plays critical roles in hydrogen peroxide production during renal fibrosis [16]. We showed that Nox4 mRNA and protein expression was significantly up-regulated in the obstructed kidney of UUO rats and markedly reduced by IMD gene transfer. By contrast, control plasmid had little effect on Nox4 expression (Figure 3). This result indicates that IMD treatment effectively inhibits Nox4 expression induced by UUO.

### IMD inhibits NADPH oxidase activation by UUO

**Figure 4.**
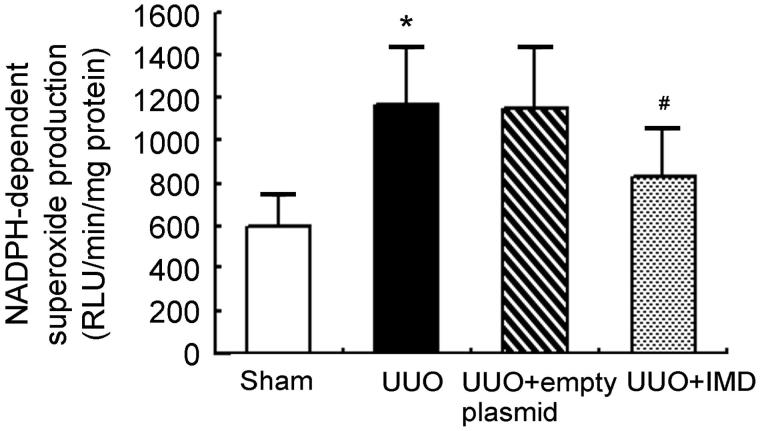
IMD attenuates UUO-induced elevation of NADPH oxidase activity. Data in bar graphs are means ± SD, *n* = 6. *p < .05 versus the sham control group; #*p* < .05 versus the UUO group.

We showed that NADPH-dependent superoxide production was significantly increased in obstructed kidney of UUO rats compared with kidney of sham controls. IMD overexpression markedly decreased NADPH oxidase activity of UUO rats. However, empty plasmid had no apparent effect on NADPH-dependent superoxide levels (Figure 4). These data suggest that IMD inhibits UUO-stimulated NADPH oxidase activation.

### IMD inhibits TGF-β1-stimulated Nox4 up-regulation and NADPH oxidase activation via cAMP-PKA-dependent pathway

**Figure 5.**
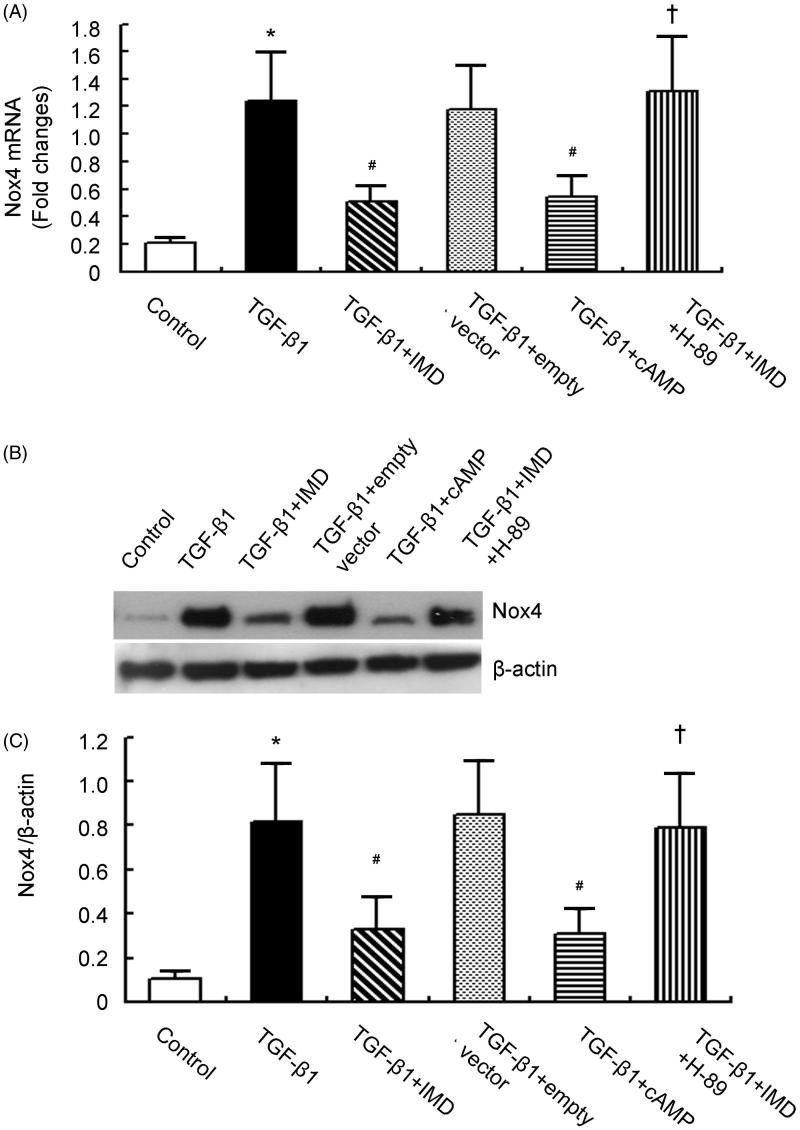
TGF-β1-induced Nox4 expression is abolished by IMD gene-transfer via PKA-dependent pathway. (A) Nox4 mRNA expression measured by quantitative RT-PCR in NRK-52E. (B) Representative Nox4 protein expression measured by Western blot in NRK-52E. (C) Densitometric quantifications of band intensities from Western blot for Nox4/β-actin in NRK-52E. Data in bar graphs are means ± SD, *n* = 6. *p < .05 versus control group; #*p* < .05 versus the TGF-β1 group; †*p* < .05 versus the TGF-β1 + IMD group.

**Figure 6.**
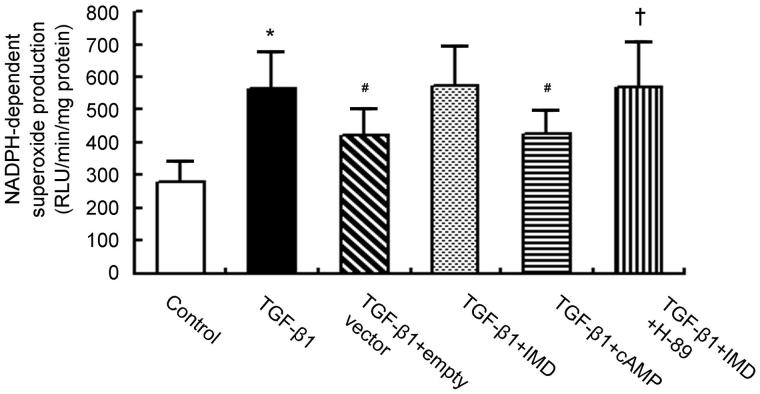
TGF-β1-simulated NADPH oxidase activation is abolished by IMD gene-transfer via cAMP-PKA-dependent pathway. Data in bar graphs are means ± SD, *n* = 6. *p < .05 versus the control group; #*p* < .05 versus the TGF-β1 group; †*p* < .05 versus the TGF-β1 + IMD group.

To determine whether the suppressive role of IMD on Nox4 expression and NADPH oxidase activation is mediated via the cAMP-PKA-dependent pathway, we tested the effects of cAMP-related compounds in TGF-β1-stimulated rat proximal tubular cell line NRK-52E. NRK-52E treated with TGF-β1 showed marked increase in Nox4 expression and NADPH oxidase activity compared to control cells. Consistent with the *in vivo* results, IMD gene delivery significantly reduced TGF-β1-stimulated Nox4 up-regulation and NADPH oxidase activation, and this effect was mimicked by cAMP analog dibutyl-cAMP (10^−3 ^mol/l). By contrast, the inhibitory effect by IMD on Nox4 expression and NADPH activity was completely abolished by H-89 (10^−5 ^mol/l), a PKA inhibitor (Figures 5 and 6). This results demonstrate that IMD inhibits Nox4 up-regulation and NADPH oxidase activation via cAMP-PKA-dependent pathway.

## Discussion

In the present study, we showed that IMD gene transfer inhibited ROS production, Nox4 expression and NADPH oxidase activity. The beneficial effects were abolished by pretreatment with PKA inhibitor H-89, and mimicked by cAMP analog dibutyl-cAMP.

Elevated ROS is a major cause of the progression and maintenance of renal fibrosis [17]. Our previous study indicated significant interstitial collagen deposition in the obstructed kidney of UUO rat [5]. Kidney-specific IMD gene delivery significantly ameliorated UUO-mediated collagen accumulation and the fibrosis-related phenotypic alterations [5]. We showed herein that IMD over-expression reduced parameters of ROS such as increased DHE generation and elevated 4-HNE staining. These may explain its anti-fibrotic effect we previously reported [5].

NADPH oxidases are reported to be important sources of ROS involved in oxidative stress [6]. Unfortunately, currently available Noxs inhibitors appear to have low specificity and they are toxic for clinical use [18]. Of the Nox isoforms, the Nox4 homolog is recognized as a principal source of ROS generation and subsequent redox-dependent pathological processes in progressive fibrotic disease [6]. The selective inhibition of Nox4 appears to be a promising approach, with the potential to be far more efficient than non-selective scavenging of ROS [19]. Nox4 has constitutive activity and consequently the overall ROS output of Nox4 may be directly governed by its expression level [20,21]. Therefore, we determined the effect of IMD on the expression of Nox4 and NADPH oxidase activation. Our results demonstrated that UUO increased Nox4 expression and NADPH oxidase activity; however, these were markedly blocked by IMD gene delivery. These results indicate the inhibitory effect of IMD on Nox4 up-regulation and NADPH oxidase activation may account for the mechanism of its potent antioxidant action on renal fibrosis.

Although Nox4 is a possible molecular target of the antioxidant action by IMD, the cellular signaling pathway remains to be determined. Because the cAMP-PKA pathway is an important postreceptor signal transduction pathway of IMD [22,23], we next examined the involvement of the cAMP-PKA pathway in the inhibitory mechanism of IMD on Nox4 expression and NADPH oxidase activity. Our present study showed that a PKA inhibitor H-89 completely abolished the beneficial effect of IMD on TGF-β1-stimulated Nox4 up-regulation and NADPH oxidase activation, and a cell permeable cAMP analog dibutyl-cAMP mimicked the effect of IMD. This result demonstrates that IMD inhibits Nox4 expression and NADPH oxidase activity through activation of cAMP-PKA pathway.

Although our current study provided significant results, there are still some limitations. The primary one is that we did not investigate the effect of IMD gene knockdown on renal ROS production and fibrosis. While the pathophysiological role of epithelial-to-mesenchymal transition of tubular cells in renal fibrosis is controversial, there is strong evidence that tubular cells play an instrumental role in orchestrating fibrosis by multiple mechanisms [24]. Our previous study had demonstrated that IMD expression was induced in the kidney, especially in tubular epithelial cells, by renal fibrosis. Thus, conditional depletion of IMD from tubular epithelial cells may provide more reliable *in vivo* evidence for a protective role of IMD against fibrosis, and requires future investigation.

## Conclusions

Collectively, our findings show that the anti-oxidant effect of IMD is mediated by downregulation of renal Nox4 expression and inhibition of NADPH activity. Nox4 is one of the major molecules targeted by IMD in exerting its anti-fibrotic effects. These effects are mediated via cAMP-PKA pathway.
